# Disulfide Bonding in Neurodegenerative Misfolding Diseases

**DOI:** 10.1155/2013/318319

**Published:** 2013-08-01

**Authors:** Maria Francesca Mossuto

**Affiliations:** Ospedale San Raffaele, Via Olgettina 60, 20132 Milan, Italy

## Abstract

In recent years an increasing number of neurodegenerative diseases has been linked to the misfolding of a specific protein and its subsequent accumulation into aggregated species, often toxic to the cell. Of all the factors that affect the behavior of these proteins, disulfide bonds are likely to be important, being very conserved in protein sequences and being the enzymes devoted to their formation among the most conserved machineries in mammals. Their crucial role in the folding and in the function of a big fraction of the human proteome is well established. The role of disulfide bonding in preventing and managing protein misfolding and aggregation is currently under investigation. New insights into their involvement in neurodegenerative diseases, their effect on the process of protein misfolding and aggregation, and into the role of the cellular machineries devoted to disulfide bond formation in neurodegenerative diseases are emerging. These studies mark a step forward in the comprehension of the biological base of neurodegenerative disorders and highlight the numerous questions that still remain open.

## 1. Introduction

*Neurodegenerative misfolding diseases* (NMD) are a group of diseases involving the misfolding of one or two proteins and their accumulation into aggregated species toxic to neurons, leading to a wide range of neurological symptoms (Table 1). Among these are Alzheimer's disease (AD), Parkinson's disease (PD), Huntington's disease (HD), prion-related disorders (PrDs), and amyotrophic lateral sclerosis (ALS). In each case a specific protein loses its functional structure to populate partially unfolded species that reorganize themselves into polymeric structures with different degrees of ordered structure, from oligomers to amyloid fibrils [1].

The fate of a protein depends on two major factors, its sequence and its cellular environment. From the sequence perspective, many studies have identified several features of the amino acid *sequence* of a protein that help predict its aggregation behavior, such as charge, hydrophobicity, patterns of polar and nonpolar residues, and tendency to form secondary structures [2, 3]. After peptide bond, the disulfide bond is the most common covalent link between amino acids in proteins. Disulfide bonds are known to stabilize proteins thermodynamically by decreasing the entropy of the unfolded state, to increase mechanical stability and to confine conformational changes [4]. From the cellular environment point of view, instead, in order to be formed, disulfide bonds need a highly efficient network of *enzymes* in particular cellular compartments, such as protein disulfide isomerases (PDIs), whose role has been revealed as central in many neurodegenerative disorders, being upregulated in many NMD and in some cases interacting directly with misfolded and aggregated proteins [5, 6]. 

Disulfide bonds are present in 15% of the human proteome and they are enriched in secreted proteins (65%), due to the need of greater protein stability in the absence of the accurate quality control systems present inside the cell. Interestingly they are present in 55% of the proteins involved in pathologic amyloid formation [7], suggesting an important role of disulfide bonds in the kinetics of aggregation and in the structure and toxicity of the formed aggregates. Do disulfide bonds have a role in neurodegenerative misfolding disorders? The analysis of a list of proteins that misfold and aggregate in neurodegenerative diseases (Table 1) revealed that only 25% of the proteins listed have disulfide bonds, a value closer to the general presence of disulfide bonds in the proteome (15%). 

**Figure 1:**
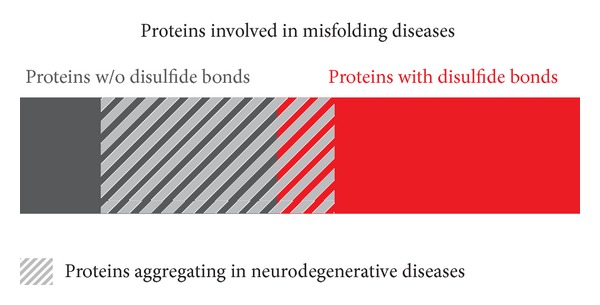
Disulfide presence (red) in proteins involved in misfolding diseases (solid) and in neurodegenerative misfolding diseases (patterned).

This distribution of disulfide bonds presence in proteins involved in misfolding diseases (Figure 1) shows that while proteins without disulfide bonds mainly aggregate in and impair neurons, proteins with disulfide bonds are mostly involved in systemic pathologies. Do neurons produce less disulfide-bonded proteins? Are neurons more sensitive to intracellular aggregates formed by cytosolic proteins (mostly disulfide free) or to the stress caused by their presence? Interestingly, a well-known cytosolic and neurotoxic protein (huntingtin fragment with extended polyglutamine repeat) has been expressed in the endoplasmic reticulum, and surprisingly it did not form aggregates [8]. The authors suggest that polyglutamine aggregation is a property restricted to the nucleocytosolic compartment and that compartment-specific cofactors promoting or preventing the aggregation of pathological proteins could exist. Is there any difference in the aggregation and cytotoxic behavior of cytosolic and secreted proteins?

The questions about protein aggregation, disulfide bonds, and neurotoxicity are numerous, and the proposed answers are often controversial. Here we focus on two simple aspects of disulfide bonds and neurodegenerative diseases: the role of disulfide bonds in the stabilization of proteins involved in aggregation and neurodegeneration and the involvement of PDIs in neurodegenerative misfolding diseases. 

## 2. Disulfide Bonds in Proteins Aggregated in Neurodegenerative Diseases

Proteins have evolved several structural and sequence-based strategies to avoid misfolding and potentially toxic aggregation such as stabilizing protein folding, controlling aggregation by gatekeepers residues, limiting *β*-propensity, hydrophobicity, and net charge [9].

Many studies on the effects of disulfide bonds on protein misfolding and aggregation revealed that (i) some disulfide bonds are positioned to prevent the population of aggregation prone conformation of some proteins (insulin, IAPP, *β*-lactoglobulin, and lysozyme) [3, 7, 10], (ii) disulfide may promote specificity in intermolecular association occurring by domain swapping [11], (iii) considering large protein structure datasets, regions of proteins involved in protein-protein interactions, and therefore often aggregation prone, was observed to be enriched in disulfide bonds [12], and (iv) in the human proteome, disulfide bonds are associated with sequences (both intra- and extracellular) of higher aggregation propensity [7]. These observations suggest that disulfide bonds have coevolved with protein sequences to minimize protein misfolding and propensity to form potentially toxic aggregates. 

The involvement of disulfide bonds in proteins involved in neurodegenerative misfolding diseases is limited to the few proteins that have them, that is, prion, SOD1, and Tau. Nonetheless, the study of the role of disulfide bonds in protein stability and in the aggregation process has not led to a definitive scenario. Here, an overview of the open questions about the involvement of disulfide bonds in each one of the tree proteins involved in NMD is presented.

### 2.1. Prions

Prion-related disorders are neurodegenerative diseases characterized by the conversion of the prion protein PrP from its normal cellular structure (PrP^C^) to a highly *β*-sheet, protease-resistant, scrapie conformation (PrP^Sc^). The only PrP disulfide bond (Cys179–Cys214) stabilizes the overall fold of the protein by connecting the two long C-terminal helices [13]. Whether the presence of the intact disulfide bond is required or not [14, 15] for aggregation, whether disulfide reshuffling occurs during PrP^C^ to PrP^Sc^ conversion *in vivo,* and whether intermolecular disulfide bonds play a role in stabilizing PrP^Sc^ aggregates [16] are still subject of some controversy [17].

### 2.2. SOD1

Amyotrophic lateral sclerosis (ALS) is the most common degenerative disease of the motor neuron system. The cause of ALS is unknown although 5–10% of cases are familial with a Mendelian pattern of inheritance [18]. About 20% of familial ALS cases share a mutation in the copper/zinc superoxide dismutase 1 (SOD1) gene [19]. SOD1 is a powerful antioxidant protein that metabolizes oxygen radicals that are produced by cellular metabolism. The active enzyme is a homodimer: each subunit contains four Cys residues at positions 6, 57, 111, and 146. An intramolecular disulfide bond between cysteine residues 57 and 146 is required for folding and stabilization of mature SOD1 [20]. Many familial ALS mutations render SOD1 more sensitive to intramolecular disulfide bond reduction [21], decreasing the apparent melting point below physiological temperature [22] and a corresponding increase in the population of their (A4V, L38V, G93A, L106V) unfolded states [23]. 

Intermolecular disulfide bonds have been identified in SOD1 aggregates in animal models of familial ALS [24, 25]. Experimental evidence suggested that even if disulfide cross-linking is not required for aggregation of mutant SOD1 [26], disulfide scrambling by intra- and intermolecular isomerization constitutes an important pathway for the aggregation of mutant SOD1 [27].

Despite the importance of disulfide bonds in SOD1 and ALS, it is not known how the intramolecular disulfide bond is formed in the reducing environment of the cytosol and how the thiol-disulfide status of SOD1 changes in the course of the disease.

### 2.3. Tau

Tauopathies are a group of neurodegenerative diseases, including AD, Pick disease, and corticobasal degeneration, characterized by the accumulation of insoluble Tau fibrils. Tau exists as six different isoforms that result from alternative splicing of mRNA [28], and the longest isoform of Tau has two Cys residues at positions 291 and 322 (numbering based upon the longest form) that can form both intra- and intermolecular disulfide bonds. Although several studies suggest that intermolecular disulfide bonds can promote Tau aggregation *in vitro*, there are lines of evidence that intramolecular disulfide bonds retard Tau aggregation *in vitro* [29]. The precise mechanisms underlying these observations remain unclear. Recent data suggest that intra- and intermolecular disulfide bonds could be one of the factors determining the range of pathological Tau isoforms [30].

### 2.4. Use of Disulfide Engineering in A*β*

In many cases a disulfide bond has been added to proteins that misfold and aggregate in neurodegenerative disorders. The most studied case is A*β*, where disulfide bonds have been engineered to allow the homogeneous population and stabilization of specific aggregated species (hairpin conformations, dimers, oligomers, and fibrils) and the subsequent characterization of their biochemical, structural, and biological properties [31]. 

## 3. Disulfide Bonding in Neurodegenerative Misfolding Diseases

In eukaryotic organisms enzyme-catalyzed disulfide bonds are formed in specialized cell compartments, such as the endoplasmic reticulum (ER) and the intermembrane space of the mitochondria [32].

The ER not only provides an environment suitable for disulfide formation, being very oxidizing with a low GSH : GSSH ratio, but it contains also many proteins dedicated to protein folding and correct disulfide bonding. Already during their translocation into the ER lumen, proteins are oxidized by protein disulfide isomerases (PDIs). The PDIs are maintained in an oxidized state by the flavoprotein Ero1, which transfers electrons directly onto oxygen generating H_2_O_2_ [33]. 

PDI was the first enzyme characterized to catalyze thiol-disulfide exchange reactions in the ER, but in recent years the number of PDI family members has been growing very quickly [34]. In humans the PDI family currently has 20 defined family members, such as ERp57, ERp72, P5, ERp44, PDIp, ERp29, ERp19, and ERdj5, defined by similarity to PDI and localization in the ER. They all contain at least one domain that is similar to one of the four thioredoxin domains of PDI, but a detailed characterization of their specific physiological function has not yet been performed. 

PDIs are a class of proteins activated upon ER stress [35]. Since extensive protein misfolding and aggregation have been found to induce massive ER stress, the involvement of PDIs in neurodegenerative misfolding diseases has been the subject of intensive studies. PDI family members have been reported to be upregulated in many mouse models of protein misfolding diseases and in postmortem human samples from patients affected with neurodegenerative diseases [5, 6]. Particular attention has been given to the role of PDIs to the pathogenesis of many neurodegenerative diseases, including Alzheimer's disease (AD), Parkinson's disease (PD), Huntington's disease (HD), prion-related disorders (PrDs), and amyotrophic lateral sclerosis (ALS). In some cases, moreover, PDIs have been also shown to directly interact with misfolded or aggregated proteins [36–39]. The results have been extensively described in recent reviews [5, 6, 40]. Here the major findings are reported and classified for pathology, to highlight which are the common features and the unclear aspects.

### 3.1. Prion-Related Disorders

Creutzfeldt-Jacob (CJD) disease was the first human brain disease shown to be associated with an upregulation of PDIs, both by a proteomic study of sporadic CJD brain tissue [41] and by the analysis of several human brain samples from patients affected with sCJD and vCJD [42]. In particular ERp57 levels correlate with the levels of prions misfolding and inversely correlate with the extent of neuronal damage in murine models of infectious scrapie prions [38, 43] suggesting that ERp57 prevents aggregation and neurotoxicity of prion protein [38].

Moreover, PDI can physically interact with misfolded prion [38, 39], and general inhibition of PDI activity with bacitracin causes the increase of aggregated prion species [39], consistently with the idea that intermolecular disulfide bond formation is also an important factor in prion misfolding [16].

### 3.2. Amyotrophic Lateral Sclerosis (ALS)

Many studies report a link between PDI and the pathogenesis of ALS. PDI and ERp57 were identified as the two main proteins upregulated in the spinal cord of a symptomatic ALS transgenic mice [44], and in the spinal cord and CSF of human sporadic ALS patients [45, 46] where PDI also colocalizes with abnormal protein inclusions associated with sporadic ALS [47]. Interestingly, PDI colocalizes with inclusions in motor neurons of mutated SOD1 (G93A) mice [44] and in human ALS patients [45]. The finding that PDI overexpression in cell culture protects against mutant SOD1 neurotoxicity [48] further indicates that PDI has an important role in protection against mutant protein aggregation in ALS. Knocking down PDI increased the levels of mutant SOD1 aggregation, and its overexpression had the opposite effect [48]. Moreover, a small molecule mimicking the active site of PDIs decreases mutant SOD1 aggregation *in vitro* [48].

Besides physically interacting with SOD1 [44], moreover, PDI interacts also with FUS [49] and colocalizes with TDP43 in human ALS tissue [47]. 

Despite its demonstrated protective role, increased levels of PDI in ALS were not beneficial in patients. As previously shown for Parkinson's and Alzheimer's brain tissues [50], posttranslational modification of PDI by S-nitrosylation of the critical active site cysteine residues, leading to the inhibition of PDI enzymatic activity, has been observed also in spinal cord tissues of sporadic ALS patients and in transgenic SOD1G93A mice. PDI was found enriched and nitrated in the aggregates isolated from the spinal cord of an ALS mouse model [36, 51]. These modifications could explain the loss of protection by PDI in disease [48].

### 3.3. Parkinson's Disease (PD)

Increased expression of PDIs family members has been observed in many studies on PD. PDI and ERp57 were found to be upregulated in two gene profile studies of PD cell culture models [52, 53]. PDIp is induced in a toxicological mouse model of PD and in brain tissue derived from PD patients [54]. PDI is overexpressed in *α*-synuclein transgenic mice [55]. The function of PDIs in PD *in vivo*, however, is still not clear.

PDI decreases the aggregation of the Parkinson's disease-associated synphilin-1 protein in neuroblastoma cells [50]. It also prevents the aggregation of *α*-synuclein in cell-free *in vitro* systems [56].

### 3.4. Alzheimer's Disease (AD)

PDI levels are increased in the brain of AD patients, where PDI colocalizes with neurofibrillary tangles in cells containing neurofibrillary tangles [57, 58]. ERp57 is found in the cerebrospinal fluid of AD patients physically associated with A*β* [37], suggesting a role as a carrier protein that prevents the aggregation of the A*β* peptide.

## 4. Conclusions

**Figure 2:**
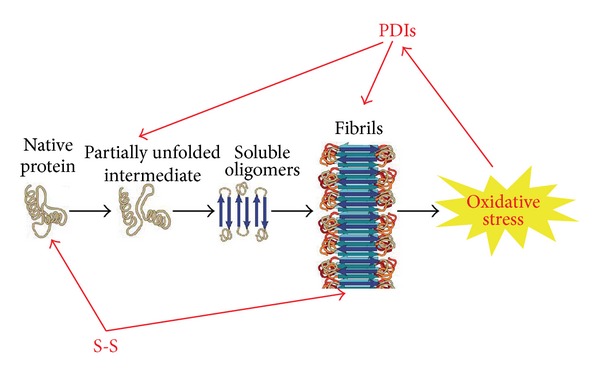
Schematic representation of the role of disulfide bonds (S-S) and disulfide bonding enzymes (PDIs) in the misfolding and aggregation of proteins involved in neurodegenerative misfolding diseases. Disulfide bonds stabilize the monomeric protein slowing down the population of aggregation-prone conformations. They also mediate in many cases the formation of aggregates by intermolecular disulfide bonds. PDIs, instead, are upregulated in the presence of protein aggregation as a general response to cellular stress and UPR activation. In some cases PDIs also interact specifically with the aggregating proteins or the aggregates. (Adapted from [1]).

Protein sequences have used different strategies to evolve and reach their functional folding in an efficient way, minimizing the risks of misfolding and (toxic) aggregation [9]. Disulfide bonds carry out this job by a double action: on one side, due to their covalent nature, they stabilize protein structure and functionality, on the other side a family of proteins (PDIs) has evolved, primarily designed to assist disulfide bonding but eventually devoted to promote protein folding and minimize protein misfolding, aggregation, and cell toxicity (Figure 2).

Intramolecular disulfide bonds stabilize the monomeric folding of prion, SOD1, and Tau retarding their aggregation. Intermolecular disulfide bonds, instead, have a more controversial role in pathogenesis. In many cases their presence is still under debate, although their importance in determining the biophysical and biological features of the formed aggregates and as a possible target for treatment.

Different potential roles of PDIs in neurodegenerative diseases are suggested by experimental findings:

- the upregulation of PDIs in animal models of NMD and in postmortem tissues from NMD patients can be part of the general UPR response to the ER stress driven by extensive protein misfolding in the cell;
- the colocalization of PDIs with protein aggregates suggests a chaperone activity of PDIs aimed at limiting protein aggregation itself. If this chaperone activity acts in stabilizing monomers or also in disrupting aggregates has not been addressed so far;
- the physical interaction of PDIs with the monomeric forms of several aggregating proteins indicates a role in modulation of the aggregation of specific disease-associated proteins by a direct association.

PDIs appear as a cellular strategy to avoid protein aggregation, but several questions remain unanswered. Which are the mechanisms of PDIs actions in affecting protein aggregation? And what about their effect on protein aggregates? Are different PDIs members specific for different substrates? Interestingly, some observations indeed indicate that some disease and protein specificity of PDI protection is likely to exist. PDI action, in fact, is not specific for amyloid-like aggregates: PDI overexpression inhibits the formation of Ig aggregation in a model of Russell Bodies formation [59] but does not decrease the number of inclusions formed by the variant of *α*1-antitrypsin [60].

Given the urgent need for novel therapies to treat neurodegenerative misfolding diseases, a solid understanding of disulfide bonding role in protein misfolding and aggregation could lead to the identification of new potential therapeutic targets.

## Figures and Tables

**Table 1:** Presence of disulfide bonds in proteins involved in neurodegenerative misfolding diseases.

Neurodegenerative disease	Protein	Cellular localization	Localization of deposits	Presence of SS
Prion-related disorders	Prion	Membrane-bound	Extra- and intracellular	Yes

Amyotrophic lateral sclerosis	SOD1	Cytosol	Intracellular	Yes
	TDP-43	Cytosol/nucleus	Intracellular	No
	FUS	Cytosol/nucleus	Intracellular	No

Alzheimer's disease (Tauopathies)	A*β*	Extracellular	Extracellular	No
	Tau	Cytosol	Intracellular	Yes

Parkinson's disease	Synuclein	Cytosolic, membrane-bound	Intracellular	No
	Synphilin-1	Cytoplasm	Intracellular	No

Huntington's disease	HTT	Cytosol	Intracellular	No

Spinal and bulbar muscular atrophy X-linked 1	Androgen receptor	Cytosol/nucleus	Intracellular	No

Spinocerebellar ataxias	Ataxins	Cytosol/nucleus	Intracellular	No
